# The Correlation Between Plasma Vitamin D and Blood Parameters in Prenatal Women

**DOI:** 10.3390/nu17162710

**Published:** 2025-08-21

**Authors:** Yi Cheng Hou, Jing Hui Wu, Lu Lu Zhao, Yin Guang Zhang, Chyi Huey Bai

**Affiliations:** 1 Department of Nutrition, Taipei Tzu-Chi Hospital, Buddhist Tzu-Chi Medical Foundation, New Taipei City 23142, Taiwan; rita@tzuchi.com.tw; 2 Department of Paediatrics, Taipei Tzu-Chi Hospital, Buddhist Tzu-Chi Medical Foundation, New Taipei City 23142, Taiwan; luluzhao2001@yahoo.com.tw; 3 Department of Obstetrics and Gynaecology, Taipei Tzu-Chi Hospital, Buddhist Tzu-Chi Medical Foundation, New Taipei City 23142, Taiwan; gymana9@yahoo.com.tw; 4 Department of Public Health, College of Medicine, Taipei Medical University, Taipei 11031, Taiwan; baich@tmu.edu.tw

**Keywords:** vitamin D, pregnancy, blood parameters, pregnancy outcome, 25-hydroxyvitamin D

## Abstract

**Background/Objectives**: Fat-soluble 25-hydroxyvitamin D (25-OHD) may be endogenously synthesized or obtained from dietary sources. Notably, it is crucial in calcium homeostasis, gene regulation, and immune system modulation, being even more relevant during prenatal stages, as the embryo utilizes vitamin D obtained from maternal plasma. Moreover, 25-OHD has been recently demonstrated to affect hematological parameters. We aimed to determine the correlation between maternal plasma 25-OHD levels, other blood parameters, and fetal anthropometric outcomes. **Methods**: Pregnant women attending an obstetrics and gynecology clinic during their gestation period were recruited, and data during follow-ups until the birth of their child were collected (IRB Approval Code: 07-XD-096). Data from 103 pregnant women were analyzed. **Results**: Compared to participants with normal levels, pregnant women with inadequate plasma 25-OHD levels exhibited a higher red blood cell count (4.3 ± 0.51 vs. 4.1 ± 0.42; *p* = 0.012) and lower mean corpuscular volume (86.4 ± 8.47 vs. 90.4 ± 6.74; *p* = 0.003), mean corpuscular hemoglobin (28.1 ± 3.34 vs. 29.6 ± 2.70; *p* = 0.008), plasma folate (12.6 ± 5.91 vs. 15.6 ± 5.86; *p* = 0.006), and vitamin B12 (289 ± 174 vs. 352 ± 147; *p* = 0.001) levels. Fish consumption frequency was positively associated with plasma 25-OHD levels. **Conclusions**: 25-OHD deficiency was correlated with alterations in hematological markers, plasma folate, and vitamin B12 levels. Given the high prevalence of 25-OHD deficiency in women of fertile age, government policies and healthcare professionals should emphasize vitamin D consumption adequacy in fertile women and expectant mothers.

## 1. Introduction

Vitamin D is an essential fat-soluble vitamin that can be synthesized endogenously through 7-dehydrocholesterol stores in the skin or from plant-based (ergocalciferol, vitamin D2) and animal-based (cholecalciferol, vitamin D3) dietary sources [1]. Notably, the latter is three times more effective than the former in raising plasma vitamin D levels in individuals and maintaining them for a longer period [2]. Moreover, sunlight exposure is the primary source of vitamin D; therefore, inadequate sun exposure, reduced outdoor activities, and the use of sunscreens and other sun protection can lead to low vitamin D levels. In these cases, dietary vitamin D can be the primary source, obtained mainly from oily fish, fish liver, liver, egg yolks, mushrooms exposed to sunlight, and fortified foods [3]. According to the eighth version of Taiwan’s Dietary Recommended Intakes (DRIs), the recommended dietary intake of vitamin D is 10 μg (equivalent to 400 IU, including for women in the gestation period) and 15 μg (equivalent to 600 IU) for individuals aged below 51 and 51 and above, respectively [4]. Vitamin D and its role in modulating calcium homeostasis in the human body have been well established. However, it plays an equally important role in various organs, such as the parathyroid gland, immune system, pancreas, and colon. Similarly, insulin secretion is controlled through the gene regulatory effect of vitamin D, by activating the vitamin D receptor in pancreatic beta cells [5]. The above facts highlight vitamin D’s role in immune system regulation and prevention of various chronic diseases and adverse outcomes during pregnancy [1,6].

Vitamin D adequacy is routinely assessed using plasma 25-hydroxy vitamin D (25-OHD) quantification, the most stable form of vitamin D in the bloodstream, which can be affected by both endogenous vitamin D production and dietary vitamin D [5]. Importantly, vitamin D deficiency may be defined using various criteria; according to those adopted in the Nutrition and Health Survey in Taiwan (NAHSIT), vitamin D deficiency, marginal deficiency, and adequacy are identified as 25-OHD < 20 ng/mL, 20 ng/mL ≤ 25-OHD <30 ng/mL, and 25-OHD >30 ng/mL, respectively [3]. This adequacy value is similar to that of the recommendations of the Practice Guidelines Committee of the Global Endocrine Society (≥75 nmol/L or ≥30 ng/mL) [7], which takes into account the effect of vitamin D beyond skeletal functions, where ≥30 ng/mL may be needed to impact the level of other hormones. Nonetheless, the Institute of Medicine has set a lower vitamin D adequacy value of ≥50 nmol/L (or ≥20 ng/mL) in all individuals [5].

According to the 2017–2020 cycle of the current NAHSIT, the mean plasma 25-OHD level among women of reproductive age falls within the marginally deficient range [3]. The prevalence of vitamin D deficiency in this group is 33.4–47.5%, with the highest rate observed in women aged 20–34 years [3]. Dietary assessment shows that 40–90% of Taiwanese individuals consume vitamin D below the Dietary Reference Intakes (DRIs), with the lowest intakes recorded in girls and women aged 13–44 years [3]. Regarding the worldwide prevalence of vitamin D deficiency, a recent systematic review reported that 15.7% individuals globally had plasma 25-OHD levels < 30 ng/mL, while 17.8% of women globally exhibited plasma 25-OHD concentrations < 30 ng/mL [8]. This implies that women living in Taiwan exhibit a higher prevalence of vitamin D deficiency, even when adopting the lower vitamin D adequacy criteria for plasma 25-OHD levels.

Vitamin D is crucial during each stage of human life, especially pregnancy, as the infant relies solely on a maternal plasma vitamin D source [5]. Animal studies suggest that vitamin D modulates placental inflammatory responses, limiting trophoblast invasion and thereby reducing the risk of pre-eclampsia [9,10]. Its roles in fetal brain development, lung maturation, and the preservation of normal function have likewise been documented [11].

Beyond these direct and indirect developmental effects, vitamin D influences hematological parameters: plasma levels correlate with erythrocyte count, hematocrit, hemoglobin (Hb), mean corpuscular volume (MCV), and other markers [12,13]. Deficiency is associated with reduced hematopoiesis, possibly through vitamin D’s regulation of the immune system, and thus the rate of anabolic activity and hepcidin-mediated iron recycling [13]. Pregnancy itself gradually expands plasma volume to meet growing demands and offset delivery-related blood loss. Although Hb and lymphocyte concentrations fluctuate across trimesters, MCV remains largely unchanged [14].

Reports on the association between vitamin D status and hematological markers in pregnancy are limited, and evidence linking vitamin D to fetal anthropometric outcomes is scarcer. In view of vitamin D’s diverse roles in fetal development, we explored the correlations between maternal plasma 25-OHD levels during gestation and selected blood parameters, as well as their relation to fetal anthropometric outcomes. We hope that through these findings, healthcare providers can be more aware of the factors affecting hematological outcomes and the importance of focusing on vitamin D intake during gestation.

## 2. Materials and Methods

### 2.1. Study Participant Recruitment and Data Collection

In this prospective study, pregnant women within the first and third trimesters were recruited from the Outpatient Department of Obstetrics and Gynecology, Taipei Tzu-Chi Hospital, New Taipei City, Taiwan, between November 2018 and August 2019. Inclusion criteria were the following: (1) age of 15–45 years with no major comorbidities (e.g., cancer, diabetes, cardiovascular disease); (2) live birth with available infant anthropometry; (3) completion of the food-frequency questionnaire (FFQ) with most items answered; (4) complete anthropometric and biochemical data. All participants provided written informed consent at enrolment and received general nutrition-related education during their clinic visits. This study was approved by the Institutional Review Board of Taipei Tzu-Chi Hospital (06-M04-071) and funded under grant TCRD-TPE-112-RT-9. Overall, 191 pregnant women were recruited.

On the day of recruitment, participants completed an FFQ, which was identical to the one used in the Pregnant Women Nutrition Status Tracking Program, a subsection of the NAHSIT, initiated by the Taiwan Ministry of Health and Welfare from 2017 to 2019 [3]. The questionnaire includes questions regarding general socioeconomic status, food consumption frequency, and vitamin D-related exposure (e.g., sun exposure). During subsequent clinic visits, participants’ urine was collected, and blood was drawn into blood collection tubes with anticoagulants, stored at 2–8 °C, and sent to a designated clinic for analysis. Ferritin and total iron-binding capacity were analyzed by the turbidimetric method; red blood cell (RBC) counts were analyzed using a hematology analyzer; hemoglobin values were analyzed by the sodium lauryl sulfate hemoglobin detection method; iron concentration was tested using UniCel DxC 800 (Beckman Coulter, Brea, CA, USA); plasma folate and vitamin B12 were analyzed using SimulTRAC-SNB radioimmunoassay (MP Biomedicals, Irvine, CA, USA); and plasma vitamin D was analyzed using a Elecsys^®^ Vitamin D total reagent kit (Roche, Basel, Switzerland). Urinary iodine was tested using a spectrophotometer (405 nm) to determine absorbance (HITACHI Spectrometer F-7000, Tokyo, Japan). Pregnancy outcomes and related data were obtained from medical records during outpatient follow-up until childbirth. After analyzing information completeness, 103 participants were eligible for inclusion in this study.

Vitamin D adequacy was assessed according to the plasma vitamin D concentration using the criteria defined by the National Institutes of Health, Bethesda, MD, USA [15]. A 25-OHD level of 20 ng/mL and above was deemed adequate. Body mass index (BMI) grouping was performed according to the adult BMI criteria defined by Taiwan’s Ministry of Health and Welfare as follows: (1) BMI < 18.5 kg/m^2^, underweight; (2) 18.5 kg/m^2^ ≤ BMI < 24 kg/m^2^, normal body weight; (3) 24 kg/m^2^ ≤ BMI < 27 kg/m^2^, overweight; (4) BMI ≥ 27 kg/m^2^, obese. The frequency of food group consumption was expressed as unit frequency/day.

### 2.2. Data Analysis

Data analyses were conducted using the R Foundation for Statistical Computing software, version 4.1.1. According to the variance and distribution of the data, an appropriate statistical analysis method (Student’s *t*-test or the Wilcoxon rank sum test, the Chi-squared test, or one-way ANOVA) was utilized. Results of continuous variables are presented as the mean ± standard deviation. Results of categorical variables are presented as *n* (%), with *n* indicating the number of patients in a particular group. Statistical significance was set at *p* < 0.05, with a 95% confidence interval.

## 3. Results

The baseline characteristics of the included participants, along with pregnancy outcomes, are shown in Table 1.

The BMI value of the group with adequate plasma vitamin D was significantly lower than that of the group with inadequate vitamin D (*p* = 0.037). The mean BMI of the two groups remained within the normal range. Age, the number of pregnancies at study recruitment, education level, income, and fetal outcomes (age at delivery, height, weight, and head circumference) did not differ between the two groups.

Table 2 displays the hematological parameters and nutritional markers in each group.

Upon stratification of hematological parameters by vitamin D adequacy, pregnant women with inadequate vitamin D levels had higher red blood cell (RBC) counts than those with adequate plasma vitamin D levels (*p* = 0.012). Moreover, the MCV and mean corpuscular hemoglobin (MCH) levels were higher in the group with adequate plasma vitamin D levels than in those without (*p* = 0.003 and *p* = 0.008, respectively). When analyzing hematology-related nutrient levels in the blood, the plasma folate and vitamin B12 levels were significantly higher in the group with adequate vitamin D levels than in those without (*p* = 0.006 and *p* = 0.001, respectively).

Table 3 presents the plasma vitamin D levels grouped by the exposure to vitamin D sources, whereas Table 4 displays plasma vitamin D levels grouped based on food group consumption frequency.

The cutoff values of each food group quantile are as follows (unit: times/day): eggs: Q1 = 0.00–0.57, Q2,3 = 0.57–1.00, Q4 = 1.01–2.50; dairy: Q1 = 0.00–0.14, Q2 = 0.15–0.43, Q3 = 0.44–1.00, Q4 = 1.01–3.50; mushrooms: Q1 = 0.00–0.14, Q2 = 0.15–0.29, Q3 = 0.30–0.43, Q4 = 0.44–2.00; fish: Q1 = 0.00–0.07, Q2 = 0.08–0.21, Q3 = 0.22–0.43, Q4 = 0.44–4.00. When stratified by the frequency of fish consumption, plasma vitamin D levels differed significantly between the first- and third-quartile (*p* = 0.008) values of plasma vitamin D. Differences in plasma vitamin D levels were not observed between the sun exposure groups, even when sun protection was considered, nor between groups categorized based on vitamin D supplementation or liver, egg, dairy, or mushroom consumption.

## 4. Discussion

Vitamin D levels correlate with various bodily functions, modulating various homeostasis-related activities. Here, BMI was inversely associated with vitamin D adequacy (*p* = 0.037), aligning with findings of previous studies assessing vitamin D levels in non-pregnant individuals [1,16,17] and pregnant women [18], with those with vitamin D deficiency exhibiting a higher BMI. The mechanism behind this observation is unclear; nevertheless, the current findings suggest that obesity may cause low plasma 25-OHD levels [17,19] by storing large amounts of vitamin D in adipose tissue. Specifically, as the proportion of adipose tissue increases, more vitamin D is stored in fat stores rather than circulating in the bloodstream. Because testing for vitamin D adequacy relies on plasma 25-OHD, adiposity may negatively affect this value. Furthermore, the expression of the vitamin D activator enzyme, CYP2R1, was lower in individuals with obesity. Importantly, this enzyme converts vitamins D2 and D3 to 25-OHD [17]. Therefore, lower CYP2R1 expression levels may imply that even when the exogenous and endogenous vitamin D supply is adequate, activation is less efficient in obese individuals.

The educational level of pregnant women did not significantly correlate with vitamin D adequacy. Previous studies have shown that educational level affects the nutritional status of individuals [20,21], which is inconsistent with our findings. Education in this context comprises two domains: primary education and nutrition-related education. Both of these serve as channels through which expectant mothers can make informed dietary decisions that benefit their own health and that of the fetus. One explanation for the discrepancy between our findings and existing evidence is that the participants received targeted education on pregnancy and nutritional adequacy, which may have mitigated the influence of lower general education on food choices and vitamin D status.

Here, income levels were not associated with vitamin D adequacy, differing from findings of research conducted on Irish children stratified by socioeconomic status [22]. Notably, a study in Northern Chinese women of childbearing age reported a similar result [23]. Ireland and Northern China are located farther from the equator than Taiwan is. Sun exposure is one of the major contributors to vitamin D adequacy, as the conversion of 7-dehydrocholesterol in the skin to vitamin D can only occur with exposure to sunlight [24]. Additionally, the Taiwanese government has implemented various policies supporting the health and nutrition of pregnant women, which include a monetary subsidy for clinic visits and pregnancy-related tests (such as gestational diabetes mellitus and anemia) during the gestation period, counseling for pregnancy nutrition, and premature birth prevention [25]. This can potentially explain the lack of differences between income statuses. Policies supporting maternal health and nutrition can help pregnant women gain access to various health facilities and make informed health-related decisions, thereby affecting their nutritional status.

Vitamin D deficiency has been associated with various adverse outcomes in pregnancy, including preterm birth, fetal death, low birth weight, and neonatal hypocalcemia [24]. These adverse effects of vitamin D deficiency are similar to what was reported in a recent umbrella review, which found that in observational studies, low levels of vitamin D (defined as < 50 nmol/L or <20 ng/mL) increased the risk of preterm birth, miscarriage and its recurrence, small-for-gestational-age, gestational diabetes mellitus, and bacterial vaginosis [26]. Differences in infant anthropometric measurements (including birth weight and head circumference) between those with adequate and inadequate circulating 25-OHD levels displayed inconsistent results between studies [26]. According to Gale et al., variations in head circumference in 9-year-old children correlated with maternal 25-OHD levels measured in late pregnancy [27], suggesting that the link between the anthropometric measurements of infants and maternal vitamin D may be observed later in the life of the infant. Aside from pregnancy outcomes, maternal vitamin D deficiency has also been correlated with asthma, type 1 diabetes, autism, schizophrenia, multiple sclerosis, and other chronic diseases in the later life of offspring [28]. This can significantly impact the quality of life of individuals. Future studies should investigate the impact of the severity of maternal vitamin D deficiency on pregnancy outcomes. Additionally, while this study may observe the impact of vitamin D deficiency at levels below <20 ng/mL, numerous studies have suggested that the optimal effects of vitamin D can be observed at levels above 30 ng/mL [29]. These effects on the human body include the musculoskeletal system and the immune system.

When comparing the hematological parameters between pregnant women in the adequate and inadequate vitamin D groups, the RBC count was higher in the latter group, while MCV and Hb values were significantly lower. Notably, the inverse association between RBC count and plasma 25-OHD levels contrasts with findings of previous studies of non-pregnant individuals, which, however, have been inconsistent [30,31]. Notably, while low vitamin D levels are positively correlated with eryptosis [30], the association of plasma vitamin D and RBC count is an inverted U shape [31]. This explains our observation that in a specific range of vitamin D deficiency, RBC count is higher than that in those with normal vitamin D levels. Vitamin D supports the biological process of RBC formation and proliferation by increasing the iron available for RBC formation [32]. This is reflected by the fact that MCH was higher in participants with adequate vitamin D. Nonetheless, an interventional study did not identify a significant correlation between vitamin D levels and RBC count [33]. Additionally, the low MCV observed in the vitamin D-deficient group does not corroborate previous findings of a lack of correlation between the two [12]. Other hematological parameters were not significantly different between the two groups, indicating that the differences observed were not caused by low iron stores (as assessed by iron, total iron binding capacity, and ferritin assays).

Vitamin D adequacy was not associated with the white blood cell count, aligning with the results of an interventional study [33]. Interestingly, plasma folate levels were positively associated with vitamin D adequacy; however, the difference in RBC folate between the two groups was not significant. Vitamin D regulates the expression of various genes, including those involved in folate and vitamin B12 absorption. The proton-coupled folate transporter, a proton–folate symporter on gastrointestinal brush-border membranes, is induced by vitamin D. Similarly, endocytosis of intrinsic factor-bound B12 from the gut lumen depends on calcium, whose absorption and homeostasis in the human body are chiefly controlled by vitamin D; therefore, vitamin D inadequacy may impair B12 regulation and absorption [34].

Major human sources of vitamin D include sunlight exposure, fish, eggs, fortified foods, and sunlight-exposed mushrooms. Among the participating pregnant women, only a higher frequency of fish consumption per week corresponded with increased plasma vitamin D concentrations. Sun exposure, vitamin D supplementation, and the intake of liver, eggs, dairy products, or mushrooms were not associated with vitamin D deficiency. Our findings regarding sun exposure and dairy consumption differ from those of a Swedish study that reported significant correlations in that population [35]; however, it is essential to note that this study only used a non-quantified FFQ, which makes it harder to obtain the quantity of each item consumed by the individual. Although sufficient vitamin D can be synthesized through adequate sun exposure, the lack of outdoor activity and sun exposure makes dietary vitamin D consumption imperative. Importantly, Weiss et al. reported that a vitamin D dose of 400 IU is likely not enough to reach 25-OHD levels of ≥30 ng/mL and that higher doses of vitamin D supplementation of 4400 IU or more may be needed to achieve the observable benefits [36]. While individuals with adequate levels of 25-OHD may maintain their levels if they adhere to the dietary guidelines of vitamin D, those who are deficient may benefit from higher vitamin D doses.

Vitamin D plays various important roles in expecting women, and its inadequacy may increase the risk of gestational diabetes, gestational hypertension, pre-eclampsia, small-for-gestational-age, and premature birth [37,38]. Therefore, given the high prevalence of vitamin D deficiency among Taiwanese women of fertile age and the high vitamin D inadequacy among this group, the government and healthcare providers should emphasize the importance of adequate sun exposure and dietary vitamin D intake. Healthcare providers should also be aware of the role of vitamin D in hematopoiesis activity, as well as the factors that influence its concentration in the body.

The main strength of this study is the analysis of complete participant data from gestation to pregnancy outcome, enabling assessment of correlations between vitamin D, maternal biomarkers, and pregnancy outcomes. An additional strength is the inclusion of comprehensive RBC and other nutrition-related biomarkers associated with fetal outcomes, allowing adjustment for multiple outcomes and confounders. Limitations of this study include the small sample size and the substantial exclusion of participants owing to incomplete information, which further reduced the cohort size and may have influenced results. Moreover, dietary intake was collected with a non-quantitative FFQ, preventing evaluation of the relationship between food quantity, rather than frequency, and plasma 25-OHD levels.

## 5. Conclusions

Pregnant women with inadequate vitamin D, determined by plasma 25-OHD levels, exhibited lower MCV and MCH but higher RBC counts. Folate and vitamin B12 levels were also reduced in vitamin D-deficient women. Higher BMI was associated with lower plasma 25-OHD. Among sun exposure and dietary vitamin D sources, only fish intake demonstrated a positive association with plasma 25-OHD. Given the various negative effects of vitamin D deficiency on women during gestation, adequate sun exposure and consumption of vitamin D-rich foods is important to support proper RBC. A balanced diet and adequate exercise can help improve vitamin D, folate, and vitamin B12 levels, which are impaired in vitamin D-deficient individuals. Additionally, a healthy lifestyle can prevent obesity, which negatively affects vitamin D concentration. Studies examining long-term dietary composition and quantified intake of specific food groups in relation to plasma 25-OHD levels are warranted.

## Figures and Tables

**Table 1:** Baseline data of study participants and pregnancy outcomes.

			Plasma 25-OHD Levels		
		All Patients (*n* = 103)	Adequate (*n* = 59)	Inadequate (*n* = 44)	*p*-Value
Age		35.8 ± 4.0	36.1 ± 4.1	35.4 ± 3.9	0.37
BMI before pregnancy		21.9 ± 3.6	21.3 ± 3.1	22.7 ± 4.0	0.037 ^†,^*
	Underweight	12 (11.6)	9 (15.3)	3 (6.8)	0.11 ^‡^
	Normal	69 (67.0)	42 (71.2)	27 (61.4)	
	Overweight	15 (14.6)	5 (8.5)	10 (22.7)	
	Obese	7 (6.8)	3 (5.1)	4 (9.1)	
Education					
	High school	4 (3.9)	3 (5.1)	1 (2.3)	0.51 ^‡^
	University	67 (65.0)	40 (67.8)	27 (61.4)	
	Masters and above	32 (31.1)	16 (27.1)	16 (36.4)	
Income					
	Low (TWD ≤ 30,000)	6 (5.8)	3 (5.1)	3 (6.8)	0.64 ^‡^
	Low-mid range (TWD 30,000–59,999)	29 (28.2)	19 (32.2)	10 (22.7)	
	Midrange (TWD 60,000–99,999)	49 (47.6)	28 (47.5)	21 (47.7)	
	High (TWD ≥ 100,000)	19 (18.4)	9 (15.3)	10 (22.7)	
Fetus age at delivery (weeks)		38.6 ± 1.3	38.7 ± 1.2	38.5 ± 1.3	0.42 ^†^
Baby’s height at delivery (cm)		49.8 ± 2.1	50.0 ± 2.1	49.6 ± 2.1	0.80 ^†^
Baby’s weight at delivery (g)		3053 ± 327	3078 ± 171	3021 ± 252	0.39
Baby’s head circumference at delivery (cm)		34.0 ± 2.5	34.1 ± 2.6	33.8 ± 2.2	0.91 ^†^

Student’s *t*-test unless marked; ^†^ Wilcoxon rank-sum test; ^‡^ Chi-squared test; * *p*-value < 0.05. Vitamin D adequacy is defined as adequate (≥20 ng/mL) or inadequate (<20 ng/mL). Abbreviations: BMI, body mass index; TWD, New Taiwan Dollar.

**Table 2:** Biomarkers in pregnant women stratified by vitamin D adequacy.

			Vitamin D Adequacy		
		All Patients (*n* = 103)	Adequate (*n* = 59)	Inadequate (*n* = 44)	*p*-Value
RBC Biomarkers					
	Ferritin (μg/L)	29.0 ± 26.3	29.8 ± 28.1	27.8 ± 24.0	0.82 ^†^
	RBC (10^6^/uL)	4.2 ± 0.5	4.1 ± 0.4	4.3 ± 0.5	0.012 ^†,^*
	Hb (g/dL)	12.0 ± 1.2	12.0 ± 1.1	12.0 ± 1.3	0.96
	HCT (%)	36.7 ± 3.0	36.5 ± 2.8	36.8 ± 3.3	0.67
	MCV (fL)	88.7 ± 7.8	90.4 ± 6.7	86.4 ± 8.5	0.0034 ^†,^*
	MCH (pg)	29.0 ± 3.1	29.6 ± 2.7	28.1 ± 3.3	0.0076 ^†,^*
	MCHC (g/dL)	32.6 ± 1.2	32.7 ± 1.0	32.4 ± 1.4	0.80 ^†^
	Iron (μg/dL)	77.1 ± 49.6	79.8 ± 37.6	73.5 ± 62.5	0.057 ^†^
	TIBC (μg/dL)	445 ± 102	446 ± 89	443 ± 118	0.63
WBC (unit/uL)		9821 ± 2260	9774 ± 2163	9882 ± 2408	0.81
Folate (ng/mL)		14.3 ± 6.0	15.6 ± 5.9	12.6 ± 5.9	0.0066 ^†,^*
RBC Folate (ng/mL)		696 ± 305	720 ± 281	665 ± 335	0.14 ^†^
Urinary Iodine (mg/L)		189 ± 208	196 ± 171	181 ± 252	0.22 ^†^
Vitamin D (ng/mL)		21.7 ± 6.8	26.1 ± 5.3	15.7 ± 3.1	<0.001 ^†,^*
Vitamin B12 (pg/mL)		325 ± 161	352 ± 147	289 ± 174	0.0012 ^†,^*

Student’s *t*-test unless marked; ^†^ Wilcoxon rank-sum test; * *p*-value < 0.05; vitamin D adequacy is defined as adequate (≥20 ng/mL) or inadequate (<20 ng/mL). Abbreviations: Hb, hemoglobin; HCT, hematocrit; MCH, mean corpuscular hemoglobin; MCHC, mean corpuscular hemoglobin concentration; MCV, mean corpuscular volume; RBC, red blood cell; TIBC, total iron binding capacity; WBC, white blood cell.

**Table 3:** Plasma vitamin D levels grouped by exposure to major vitamin D sources.

		*n*	Vitamin D (ng/mL)	*p*-Value
Sunlight Exposure				
Including Protected Exposure				
	No/Almost none	33	23.2 ± 8.0	0.19 ^†^
	Yes	70	21.0 ± 6.2	
Not Including Protected Exposure				
	No/Almost none	70	21.4 ± 7.2	0.53
	Yes	33	22.3 ± 6.2	
Vitamin D Supplementation				
	Yes	12	20.3 ± 6.5	0.42 ^†^
	No	91	21.8 ± 6.9	
Dietary Sources—Liver				
	Do consume	21	22.1 ± 5.2	0.33 ^†^
	Do not consume	82	21.5 ± 7.2	

Student’s *t*-test unless marked; ^†^ Wilcoxon rank-sum test; *p*-value < 0.05.

**Table 4:** Plasma vitamin D levels stratified by quartiles of major dietary vitamin D source consumption.

	Quartiles				*p*-Value
	Q1	Q2	Q3	Q4	
Eggs (times/day)	22.1 ± 7.1	20.7 ± 6.9 ^‡^		23.7 ± 6.1	0.12
Dairy (times/day)	20.4 ± 7.1	22.6 ± 6.7	21.1 ± 6.7	24.7 ± 7.5	0.42
Mushrooms (times/day)	21.4 ± 7.5	20.1 ± 5.7	23.7 ± 7.2	22.9 ± 6.7	0.32
Fish (times/day)	18.5 ± 7.3 ^a^	21.9 ± 6.6	23.9 ± 6.8 ^b^	22.6 ± 5.1	0.0076 *

Quartiles assessed using one-way ANOVA; * *p*-value < 0.05; ^‡^ repeating values in the dataset led to the lack of distinction between the two quantiles; ^a,b^ significant difference between the groups.

## Data Availability

The datasets generated during and/or analyzed during the current study are available from the corresponding author on reasonable request due to ethical and privacy considerations.

## Abbreviations

The following abbreviations are used in this manuscript:

25-OHD	25-hydroxyvitamin D
DRIs	Dietary recommended intakes
NAHSIT	Nutrition and health survey in Taiwan
Hb	Hemoglobin
MCV	Mean corpuscular volume
FFQ	Food-frequency questionnaire
BMI	Body mass index
ANOVA	Analysis of variance
RBC	Red blood cell
MCH	Mean corpuscular hemoglobin
